# Exploring diverse approaches for predicting interferon-gamma release: utilizing MHC class II and peptide sequences

**DOI:** 10.1093/bib/bbaf101

**Published:** 2025-03-11

**Authors:** Abir Omran, Alexander Amberg, Gerhard F Ecker

**Affiliations:** Department of Pharmaceutical Sciences, University of Vienna, Josef-Holaubek-Platz 2, 1090 Vienna, Austria; Sanofi, Preclinical Safety, Industriepark Höchst, 65926 Frankfurt am Main, Germany; Department of Pharmaceutical Sciences, University of Vienna, Josef-Holaubek-Platz 2, 1090 Vienna, Austria

**Keywords:** machine learning, T-cell response, immunology, predictive modeling

## Abstract

Therapeutic proteins are in high demand due to their significant potential, driving continuous market growth. However, a critical concern for therapeutic proteins is their ability to trigger an immune response, while some treatments rely on this response for their therapeutic effect. Therefore, to assess the efficacy and safety of the drug, it is pivotal to determine its immunogenicity potential. Various experimental methods, such as cytokine release or T-cell proliferation assays, are used for this purpose. However, these assays can be costly, time-consuming, and often limited in their ability to screen large peptide sets across diverse major histocompatibility complex (MHC) alleles. Hence, this study aimed to develop a computational classification model for predicting the release of interferon-gamma based on the peptide sequence and the MHC class II (MHC-II) allele pseudo-sequence, which represents the binding environment of the MHC-II molecule. The dataset used in this study was obtained from the Immune Epitope Database and labeled as active or inactive. Among the approaches explored, the random forest algorithm combined with letter-based encoding resulted in the overall best-performing model. Consequently, this model’s generalizability to other T-cell activities was further evaluated using a T-cell proliferation dataset. Furthermore, feature importance analysis and virtual single-point mutations were conducted to gain insights into the model’s decision-making and to improve the interpretability of the model.

## Graphical Abstract

**Graphical Abstract:**
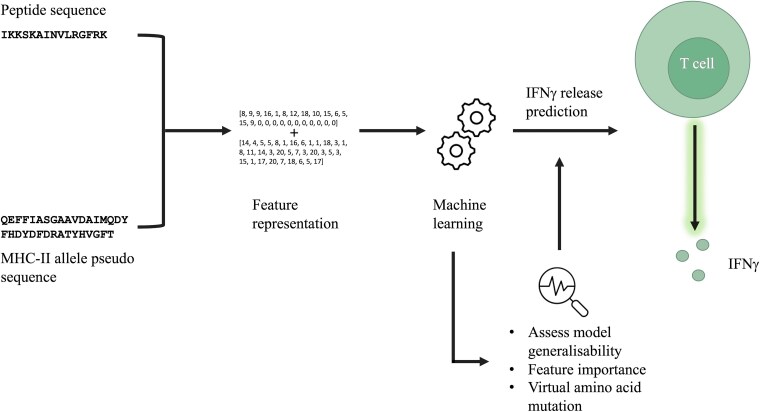

## Introduction

In 1880, the first antibody-based treatment, a serum therapy against diphtheria, was discovered by Behring [1, 2]. It was not until 1986 that the first therapeutic monoclonal antibody (mAb), Muromonab-CD3, a murine mAb, was approved for clinical use [1]. Muromonab-CD3 frequently caused severe adverse reactions, including cytokine storms, leading to its withdrawal in 2010 [3]. Today, the market size for therapeutic proteins continues to grow and is estimated to reach 47.4 billion US dollars by 2032 [4]. Therapeutic proteins are widely sought-after drugs, offering treatment for numerous acute and chronic diseases that previously were considered untreatable. Compared to small molecules, they exhibit high selectivity, which generally results in fewer off-target adverse events. Nonetheless, therapeutic proteins can still provoke an immune response, potentially leading to a variety of adverse events.

Vaccines rely on the immune response as part of their efficacy. They introduce an antigen into the body, prompting the immune system to respond to it. This process enables the body to ‘remember’ the antigen, allowing for a faster and more effective defense upon future exposure. A key component of this immunogenic response is the major histocompatibility complex (MHC) presentation pathway, which plays a crucial role in antigen recognition and immune activation. The process starts with the cleavage of the protein into peptide fragments that can potentially bind to the MHC molecules. Peptides binding to MHC class II (MHC-II) usually have a length of around 12–25 residues [5, 6]. The MHC-II molecule contains four binding grooves and specifically recognizes positions 1, 4, 6, and 9 on the peptide, which are essential for binding [5]. In addition, genetic factors influence binding, as peptides interact with different alleles with varying affinities, leading to differences in the immunogenic response [7]. Even a single-point mutation in an MHC-II molecule can significantly alter its binding affinity [8]. When a peptide binds to the MHC-II molecule, it forms a peptide–MHC-II complex (pMHC-II). Subsequently, this complex translocates to the cell surface, where it presents the peptide to a T-cell, initiating T-cell activation. Specifically, the MHC-II molecule presents the peptide to a CD4+ T-cell, also known as a T-helper cell. The peptide residues exposed to the T-cell receptor and primarily involved in binding are positions 2, 3, 5, 7, and 8 [9]. Upon activation, T-helper cells can differentiate into distinct subsets, including T-helper 1 (T_H_1) cells. These cells play a crucial role in cell-mediated immunity by secreting cytokines that activate other immune cells, such as cytotoxic T-cells (CTL). The main cytokine secreted by T_H_1 cells is interferon-gamma (IFNγ). However, an excessive pro-inflammatory response can result in tissue damage [10] or chronic inflammation [11]. Therefore, assessing a drug’s immunogenicity potential is essential to ensure both its safety and efficacy.

According to the Food and Drug Administration guidance on immunotoxicity, the most suitable assay for assessing immunogenicity risk should be selected, such as the cytokine release assay [12]. There are several methods to evaluate if a peptide has the potential to induce an immunogenic response. The ELISpot assay can be used to measure IFNγ release, providing insight into T-cell activation and the immunogenic potential of a therapeutic protein [13]. However, a cytokine release assay requires allele matching, which is crucial for accurate measurement outcomes [14]. MHC-multimer-based flow cytometry is another technique used to assess T-cell response, but due to HLA polymorphism, it is not suitable for population-wide analysis and is only effective for individuals with a specific set of MHC alleles [15]. Other methods, such as T-cell proliferation assays, require prolonged incubation times [14], making them both time-consuming and costly. Consequently, it is essential to develop methodologies capable of rapidly and efficiently screening a large set of peptides across multiple MHC alleles. In this context, *in silico* methods serve as valuable tools, enabling rapid and comprehensive analysis, which is crucial for predicting and assessing a peptide’s immunogenic potential for a given MHC allele of interest.

Several *in silico* methods exist for predicting and evaluating a peptide’s immunogenic potential. POPISK is a model that predicts whether a peptide associated with the HLA-A02:01 allele has the potential to be immunogenic based on T-cell response data [16]. Other computational tools, such as NetMHCIIpan, focus on predicting peptide binding affinity to a given MHC allele [17]. Additionally, models leveraging Bidirectional Encoder Representations from Transformers (BERT) models have been trained on a high number of protein sequences to address biological tasks, including peptide–MHC binding predictions [18, 19]. BERT models, originally developed for natural language processing, generate rich contextual embeddings from large protein sequence datasets, making them valuable for downstream analyses. Other protein descriptors, such as z-scale descriptors, are commonly used in proteochemometric models to describe the binding site environment of proteins. The descriptors characterize the amino acids’ physicochemical properties [20], providing informative input for the model.

Dhanda *et al.* developed an *in silico* method for predicting a specific cytokine release, such as IFNγ [21]. Their approach utilized binary patterns, composition, and motifs as features for the peptide sequence to predict IFNγ release. The model, however, did not account for the MHC-II allele in the prediction. To the best of our knowledge, the model proposed by Dhanda et al. is currently the only model for IFNγ release prediction. Therefore, in this study, we explored various approaches for building IFNγ release models using information from the peptide sequences and the MHC-II allele pseudo-sequences. Given the complexity of the T-cell response, we explored different approaches to enhance the model’s performance and generalizability. This involved testing various descriptors, such as letter-based encoding (LBE), ProtBert embedding features, and z-scale descriptors, to characterize both peptide and MHC-II allele sequences. In addition, we adopted an active learning (AL) approach. Beyond evaluating model performance, our study also examined model interpretability through feature importance analysis and virtual single amino acid mutation experiments in the peptide sequences.

## Materials and Methods

### Data collection and preprocessing

Data were obtained from the Immune-Epitope Database (IEDB) and filtered for a human host, MHC-II, and positive and negative assays. The pMHC-II pairs were labeled based on the majority measurement. For instance, if the pMHC-II had five measurements recorded in the database for IFNγ release, and three out of five were negative, then the pMHC-II was labeled as inactive. Pseudo-sequences representing the binding site environment of MHC-II molecules, each 34 residues in length, were used for the MHC-II alleles [22]. The dataset was further refined based on peptide length, as different studies have suggested varying optimal lengths. For this study, we selected peptides ranging from 12 to 24 residues. Lastly, all duplicates based on the peptide sequence, the MHC-II allele pseudo-sequence, and activity were reduced to a single instance, whereas duplicates based solely on the peptide sequence and the MHC-II allele pseudo-sequence were completely removed from the dataset. This resulted in an imbalanced dataset with 7266 pMHC-II pairs, where the inactive class accounted for 30% of the total data.

#### Dataset split

**Figure 1:**
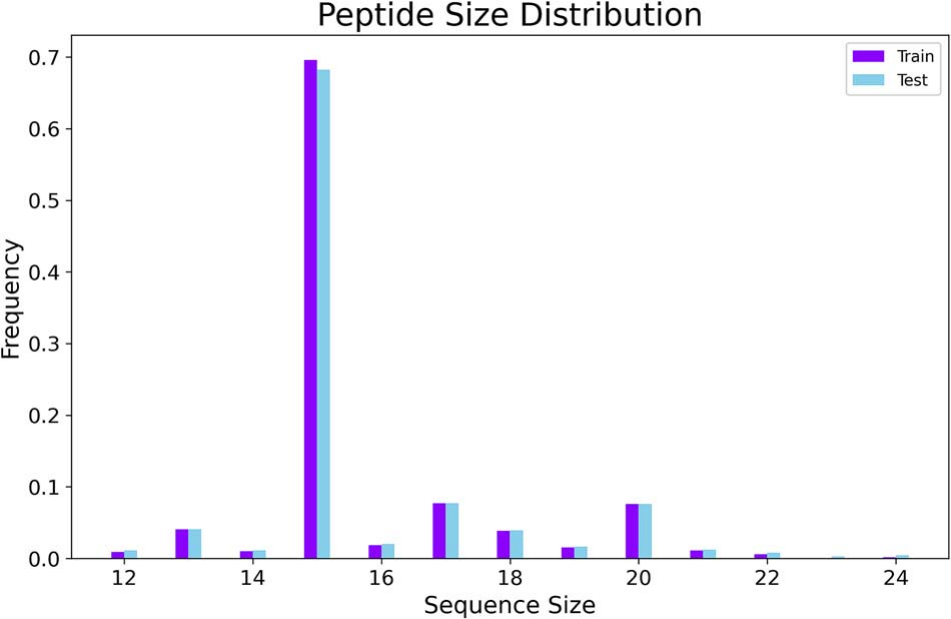
The figure visualizes the peptide sequence length distribution in the training and test set in the first fold. The majority length was 15 residues, accounting for 70% of the dataset.

A 10-fold cross-validation (CV) was performed, and given the imbalance in classes and peptide sequence lengths, stratified splitting was applied to preserve the original dataset’s distribution across all subsets. As shown in Fig. 1, the most common peptide length was 15 residues, which comprised 70% of the dataset.

### Model development

#### Representation

We explored three types of descriptors: LBE, ProtBert embedding features, and z-scale descriptors. While LBE and ProtBert features utilized peptides of various lengths, z-scale descriptors required sequences of uniform length. Therefore, we selected the majority length in the dataset (15 residues), resulting in a 30% reduction in dataset size. The z-scale descriptors were generated using the peptides library in Python. In order to enable the comparison with the LBE method, the same dataset was used, referred to as LBE 15mer.

The ProtBert model was used to generate features for the sequences in Python. Trained on 217 million protein sequences, ProtBert captures contextual information that could be beneficial for our task. [23]. These features have the potential to enhance model performance and generalizability. We started with ProtBert, as it was less complex than some of the other BERT models, allowing us to establish a clear baseline without imposing additional computational demands.

**Figure 2:**
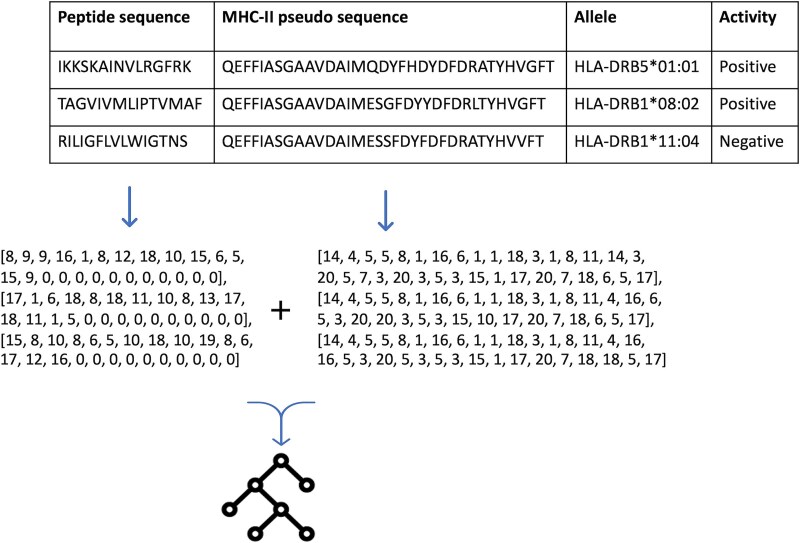
The figure depicts the data for the modeling and how the data was used as input for the LBE models. Unique numerical values representing the amino acids were used to encode the sequences. Subsequently, the LBE was concatenated and used as input for the model.

The descriptors used to describe the peptide sequence and the MHC-II pseudo-sequence were concatenated and then used as input to the model. However, before concatenating the LBE descriptors, the peptide sequences were padded with zeros until reaching the length of 25, as depicted in Fig. 2.

#### Model building

Classical machine learning algorithms, such as random forest (RF), provide greater interpretability than deep learning models. Thus, RF, support vector machine (SVM), and gradient boosting machine (GBM) were selected as the three algorithms used in this study across the three different feature representations. To address class imbalance in the dataset, the probability threshold for classifying active samples was adjusted. Rather than using the default threshold of 0.5, multiple values were tested, and the threshold that achieved a better balance between sensitivity and specificity was chosen for the final model, which was 0.65. Another approach to address the imbalanced dataset was AL [24], applied to the LBE model. For each iteration, 10 samples with the highest uncertainty were added to the training set to refine the model. This approach helps the model to learn the data distribution where it struggles most, meaning that in the case of imbalanced data, the model might gain more insight into the minority class. As a result, AL has the potential to enhance generalization. The best-performing algorithm for the LBE model was subsequently used for the LBE 15mer model and the AL approach.

#### Hyperparameter optimization

A randomized search was performed instead of a more exhaustive grid search, as it allows for a more efficient exploration of a wider range of hyperparameters. This approach provides better coverage in comparison to a grid search with the same parameter settings. The random search was performed with a 10-fold CV for all models, with the selected parameters detailed in Supplementary Tables 1–2. The same hyperparameters found for LBE were also utilized for the AL approach.

#### Model performance evaluation metrics

For evaluation of the performance of the binary models, the scikit-learn library was employed in Python to calculate various metrics. The metrics included balanced accuracy, Matthews correlation coefficient (MCC), precision, sensitivity, and specificity.

### Model assessment

#### T-cell proliferation dataset

Since no additional IFNγ release data could be identified, creating an external test set was not feasible. To address this limitation, an alternative dataset was used to evaluate if and to what extent the model could predict the outcome of a T-cell assay type closely associated with IFNγ release. Data points for T-cell assay types, such as T-cell proliferation and IL-2 release, were extracted from IEDB. After filtering out overlapping data from the IFNγ release dataset, a T-cell assay with sufficient data containing both active and inactive samples was obtained. The final T-cell proliferation dataset comprised 711 data points, with 600 active and 111 inactive samples, making it suitable for prediction. All other assay types provided a smaller dataset with very few inactive samples. The T-cell proliferation dataset was applied to the model that demonstrated the best overall performance and was used in place of a test set in the 10-fold CV, enabling a direct comparison with the performance on the IFNγ release test set.

### Model interpretability

#### Feature importance

A feature importance procedure was conducted to identify the top five most important amino acid positions. Subsequently, the amino acid frequency at these positions was examined to identify discrepancies between the two classes, helping to assess whether the model was capturing valuable information regarding T-cell response. The feature importance was only performed on the LBE 15mer model to facilitate the analysis and interpretability. The scikit-learn library was used to compute the feature importance for tree-based models.

#### Virtual peptide single amino acid mutation

A virtual single amino acid mutation was applied to each distinct amino acid at every position individually within the test set. This approach allowed for the assessment of which amino acids in specific positions had a greater impact on the model’s predictions. Therefore, the difference in error rate (ΔER) between the true prediction against the mutated prediction was used as a metric in this analysis, calculated as shown in equations (1) and (2). A ΔER value of zero indicates that the mutation had no effect on the prediction, a positive ΔER value signifies an increase in ER, while a negative ΔER reflects a decrease in the ER. For consistency with the feature importance analysis, this method was applied exclusively to the LBE 15mer model.


(1) \begin{equation*} Error\ rate=\frac{Number\ of\ incorrect\ predictions}{All\ predictions} \end{equation*}

(2) \begin{equation*} \Delta Error\ rate={Error\ rate}_i\hbox{--} {Error\ rate}_j \end{equation*}

The predictions were performed twice to calculate the ΔER, and overlapping mutations that resulted in a statistically significant ΔER within the top five positions were selected for further analysis. The top 50 mutations, the MHC alleles associated with the peptides, and the corresponding changes in activity were identified. Statistical significance was assessed using the Wilcoxon test in Python.

## Results

### Model performance

Exploring multiple approaches to predict IFNγ release from T-helper cells resulted in the development of eleven models. Among the algorithms tested, the RF consistently outperformed SVM and GBM across all representations (see Supplementary Table 3). The simplest model, built using LBE descriptors, demonstrated the best overall performance (Table 1). The z-scale model and the LBE 15mer, which used the same dataset, showed nearly identical performance. Thus, the additional information provided by the z-scale descriptors did not significantly improve the model’s performance. Meanwhile, the ProtBert model resulted in the lowest sensitivity but the highest specificity among all models. This indicates that it was less effective as the other models in capturing the active samples, but better regarding the inactive ones. The standard deviation of the different metrics presented in Table 1 shows that the different methods did not result in notable differences in stability; however, considering all the metrics, the z-scale based model appeared to be the least stable one.

**Table 1:** The table presents the model performances for all representations using a RF algorithm. The values are presented as the mean metric of the 10-fold CV, with the standard deviation indicated in parentheses. The model demonstrating the best overall performance is shown in bold. The representations used are LBE, z-scale descriptors, and ProtBert embedding features. In addition, an AL approach was applied with the LBE representation.

Method	Algorithm	Balanced accuracy	MCC	Precision	Sensitivity	Specificity
LBE	**RF**	**0.78 (0.01)**	**0.53 (0.02)**	**0.88 (0.01)**	**0.78 (0.02)**	**0.77 (0.03)**
LBE 15mer	RF	0.75 (0.01)	0.49 (0.02)	0.85 (0.01)	0.73 (0.02)	0.78 (0.03)
Z-scale	RF	0.76 (0.02)	0.50 (0.04)	0.86 (0.01)	0.74 (0.03)	0.78 (0.03)
AL (LBE)	RF	0.76 (0.02)	0.51 (0.03)	0.86 (0.02)	0.74 (0.03)	0.78 (0.03)
ProtBert	RF	0.76 (0.01)	0.50 (0.03)	0.89 (0.01)	0.72 (0.03)	0.81 (0.03)

The AL approach was applied to the LBE model to assess whether the model’s generalizability could be further improved. After 351 iterations, the model reached the highest mean MCC and was selected as the final model (see Supplementary Fig. 1). The performance across all folds is presented in Supplementary Fig. 2. While the AL model did not surpass the performance of the LBE model, the difference was minimal.

### T-cell proliferation dataset performance

**Figure 3:**
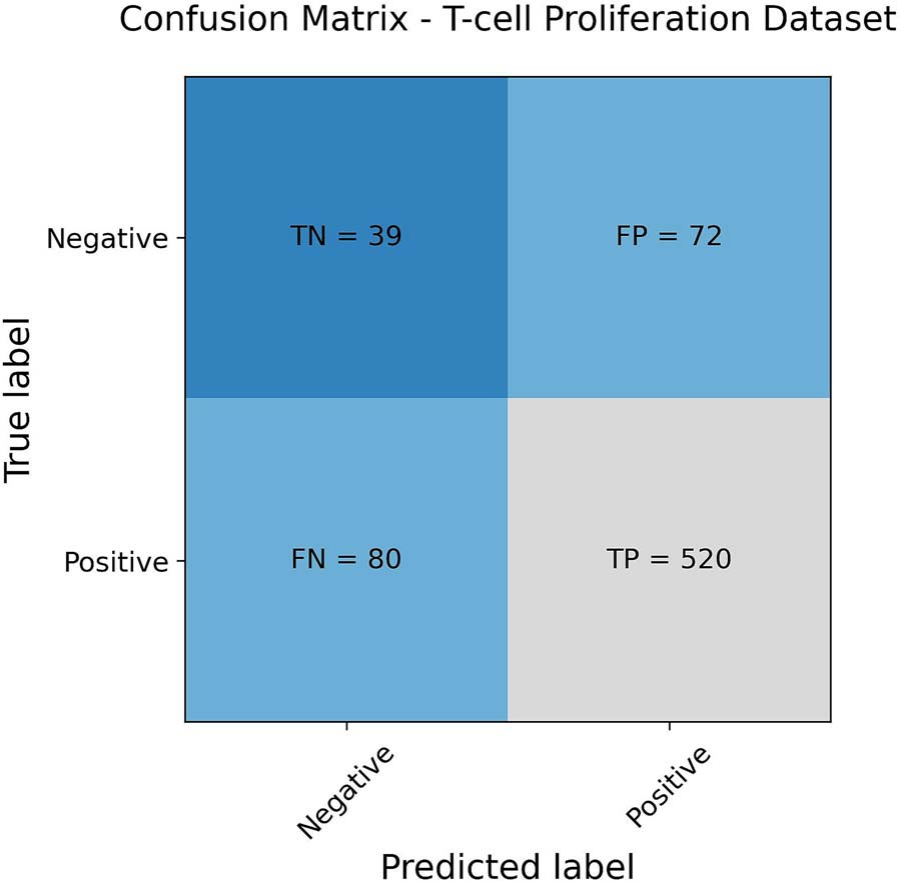
The figure depicts the confusion matrix obtained from the T-cell proliferation dataset, highlighting the high false positive rate. However, it also shows that the model was able to capture most of the active samples.

The T-cell proliferation dataset was used to assess the model’s performance. As expected, the model did not perform as well on the T-cell proliferation dataset as it did on the internal IFNγ release test set. However, it still correctly identified the majority of active samples, as presented in Table 2. Notably, the model achieved a higher sensitivity while maintaining the same precision as in the IFNγ release test set. However, the specificity results indicate that it struggled to accurately predict inactive samples. The confusion matrix in Fig. 3 illustrates the predictions for the T-cell proliferation dataset. Given that 84% of this dataset consisted of active samples, the model successfully classified most of them correctly.

**Table 2:** The result of the T-cell proliferation dataset using the LBE model is presented. The values represent the mean performance, with the standard deviation shown in parentheses.

Method	Balanced accuracy	MCC	Precision	Sensitivity	Specificity
LBE	0.61 (0.02)	0.21 (0.03)	0.88 (0.01)	0.87 (0.01)	0.35 (0.05)

### Feature importance

**Figure 4:**
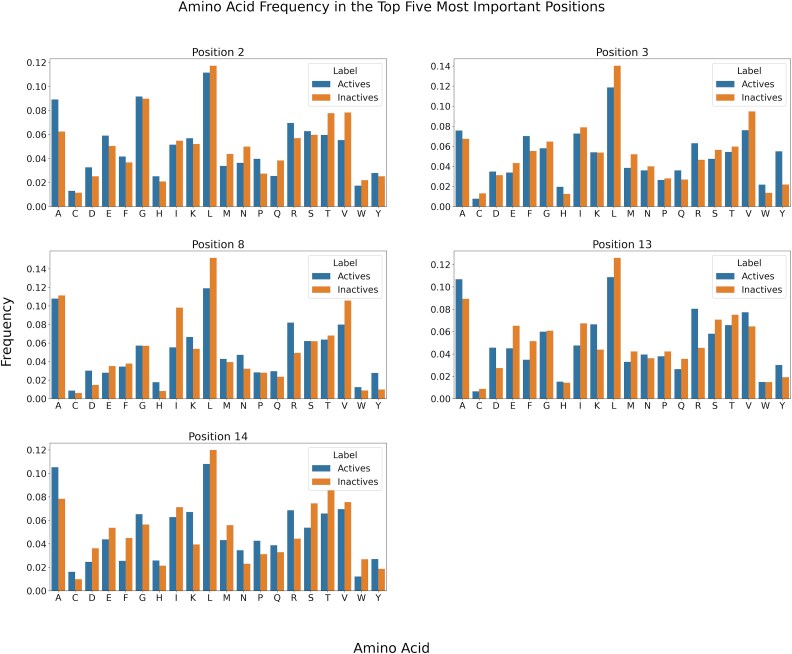
The amino acid frequency in the top five most important positions for the active and inactive classes.

The LBE 15mer model was used for the feature importance analysis to identify the most influential amino acid positions in the peptides. This analysis was conducted with the 10-fold CV, and the final top five features, determined from all results, are presented in Table 3. The most important position (P) was P3, which is known to be involved in T-cell binding. Two additional positions known to be involved in T-cell binding are P2 and P8, which also ended up among the top five features. The remaining two other positions among the top five features, P14 and P13, are unlikely to be directly involved in either MHC-II binding or T-cell binding. The amino acid frequency was analysed at the top five most important positions, revealing no notable differences between the active and inactive classes (see Fig. 4). The most frequent amino acid in all top five positions was leucine for both the active and inactive classes. Therefore, no notable discrepancies were observed in the amino acid frequency between the two classes.

**Table 3:** The top five most important features and their rank are presented in the table. The features represent the different positions (P) in the peptide sequence.

Rank	Positions
**1**	P3
**2**	P14
**3**	P2
**4**	P8
**5**	P13

### Virtual peptide single amino acid mutation

**Figure 5:**
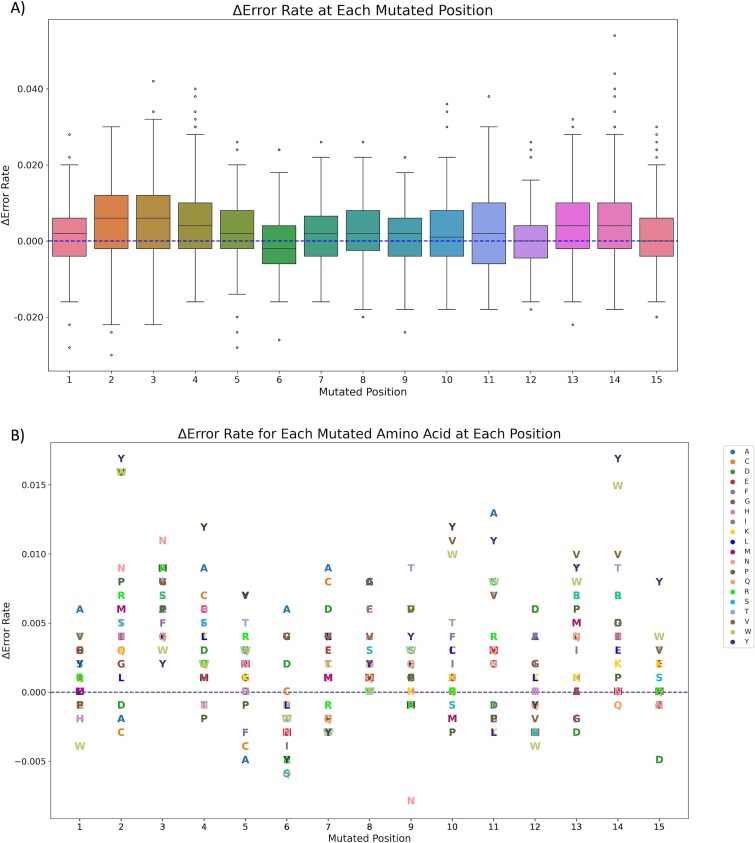
The figure depicts the impact on the prediction after the virtual single amino acid mutation in different positions using the 15mer model. The ΔER was calculated and used as a metric to evaluate the impact of the predictions, where a ΔER value of zero indicates that the prediction was not impacted by the mutation. A) The ΔER for all positions and the median ΔER are depicted in the boxes. B) The ΔER for each mutated amino acid at each position.

Further insight was acquired by evaluating how individual amino acid variations at a given position in the peptide sequence could impact the prediction. A virtual single amino acid mutation was applied to each position in the peptide sequence, and the LBE 15mer-based model was used to predict the outcomes. The results indicate that predictions were influenced by both the given amino acid and its position. Figure 5 visualizes the ΔER based on the specific mutation in the peptide sequence. The top five positions with the highest ΔER were P2, P3, P8, P13, and P14, as illustrated in Fig. 5A. Four of these positions were also ranked among the top five ones in the feature importance analysis.

A tyrosine mutation at positions P2 and P14 resulted in the highest ΔER of 0.017, followed by valine and tryptophan mutations at P2, each yielding a ΔER of 0.016. In contrast, an asparagine mutation in P9 produced the lowest ΔER observed, as shown in Fig. 5B. These findings highlight the amino acids that play a crucial role at specific positions in the peptide in influencing prediction variability. The same amino acid mutated in different positions can have varying results, as was observed for tyrosine, which obtained a negative ΔER in P6, P7, and P12, but a positive one in P2 and P14.

Furthermore, the results shown in Fig. 5 highlight only the mutated amino acid responsible for the change in prediction, without considering the original amino acid that was mutated. To address this, Supplementary Figs. 3–10 provide detailed information on the count of prediction changes associated with each original and mutated amino acid for specific MHC alleles. This analysis is presented separately for the two classes, focusing on the five most important positions identified in the feature importance analysis, as well as the mutations that resulted in a statistically significant ΔER at these positions. Table 4 lists the mutated amino acids and their positions that resulted in a *P*-value < 0.05. Notably, none of the mutated amino acids in P8 resulted in a *P*-value < 0.05. Some mutations exhibited variable effects depending on the associated MHC allele, meaning they could shift the prediction from active to inactive or vice versa. For instance, when alanine, aspartic acid, and glycine were mutated to tyrosine, the resulting prediction varied between active and inactive depending on the MHC allele linked to the peptide. Specifically, tyrosine, which resulted in the highest ΔER in P2, caused a change in prediction from active to inactive when replacing glycine in peptides associated with MHC alleles HLA-DRB10901 and HLA-DRB10102 (see Supplementary Fig. 3). However, as shown in Supplementary Fig. 7, the same mutation in inactive peptides resulted in a change when the peptide was associated with MHC allele HLA-DRB10802.

**Table 4:** The mutated amino acids that resulted in a statistically significant change in ΔER in the positions ranked among the top five most important features are presented. The mutated amino acids in P8 did not result in a statistically significant ΔER.

Position	Mutated amino acids
P2	R, Y, W, T, V
P3	K, N, Q
P13	R, N, P, T, S, V, Q
P14	Y, G, W, T, S, V

## Discussion

This study focused on building IFNγ release models using information from peptide sequences and MHC-II alleles pseudo-sequences. Furthermore, various descriptors were explored to describe the amino acid sequences. Information-rich descriptors, such as z-scale and ProtBert embedding features, were compared to the most straightforward method, the LBE model. The z-scale descriptors capture physicochemical properties, such as hydrophobicity, steric, and electronic properties. In contrast, the ProtBert model, trained on a large set of protein sequences, identifies complex patterns within amino acid sequences, providing a more biologically meaningful representation. Nonetheless, considering the overall model performance, the models trained on these information-rich descriptors did not outperform the LBE models. One possible explanation for the lack of significant improvement could be due to the increased input dimensionality. Both the z-scale and ProtBert features expand the complexity of the input space, potentially making it more challenging for the model to effectively learn from the information. Another factor that complicates the model learning process is the imbalanced dataset, which can reduce generalizability. To address this, we applied an AL approach to the LBE model, as it demonstrated the best performance. AL has the potential to increase the model’s performance or improve generalizability while using fewer data points. This was achieved by selecting the top 10 data points with the highest uncertainty for each iteration and adding them to the training set. However, also this strategy did not improve the model’s performance or its ability to correctly predict more inactive samples. As a result, the model remained limited in its capacity to capture the inactive class, which represents the minority class in the dataset. In addition, as shown in Supplementary Fig. 2, the model’s performance did not improve with additional iterations beyond 351. The mean MCC value fluctuated within a narrow range of 0.49–0.51, suggesting that the model reached a learning plateau.

Predicting T-cell response is a highly complex task involving multiple steps. However, the models demonstrated sufficient performance for this task. To further assess their applicability, we investigated whether the best-performing model, the LBE model, could predict an activity closely related to IFNγ release. For this purpose, the model was applied to the T-cell proliferation dataset. The prediction resulted in a precision and sensitivity close to 0.90, indicating that the model was very good at capturing active samples. However, it struggled with correctly predicting inactive samples. Given that only 16% of the T-cell proliferation dataset consisted of inactive samples, the evaluation metrics for the minority class may be skewed and may not fully reflect the model’s ability to capture inactives. Nonetheless, the model achieved a balanced accuracy of 0.61. Considering that the T-cell proliferation dataset is highly imbalanced and represents a slightly different endpoint than the dataset used to build the model, this result suggests that the model was able to capture meaningful information related to a T-cell activity and generalized to a certain extent to the related but distinct dataset.

To further examine and better understand which information was captured and learned by the model, a feature importance analysis was performed. Positions P2, P3, and P8 were identified among the top five most important features, aligning with positions known to be involved in T-cell binding [9]. Additionally, positions P13 and P14 were ranked among the top five positions, despite not being directly involved in MHC-II or T-cell binding. However, these positions can still impact the affinity for the MHC-II molecule as well as the stability of the pMHC-II complex, as demonstrated in various studies [25–27]. Therefore, the specific amino acids at these positions affect the stability of the complex, which in turn influences the outcome of the T-cell response. These findings suggest that the model successfully captured biologically relevant information. To further explore potential differences between active and inactive samples, the amino acid frequency at the top five positions was analysed. No significant discrepancies were observed, indicating that factors beyond the presence of a single amino acid at a given position contribute to the observed activity. Subsequently, we conducted a virtual single amino acid mutation at every position to assess the impact on the model’s predictions. Four of the positions with the highest feature importance also exhibited the highest median ΔER values, suggesting that mutations at these positions were more likely to impact the prediction. However, the results also revealed that the effect varied depending on the specific amino acid, indicating that prediction changes were not solely determined by the position but also influenced by the amino acid itself.

Sant’Angelo et al. showed that an alanine mutation in P2 led to an increased T-cell response [9]. In our analysis, five amino acids at position P2 showed a statistically significant ΔER, indicating that mutations at this position influenced the model’s prediction. This suggests that the model may associate these mutations with a potential change in T-cell response. Furthermore, the results showed that, depending on the MHC allele, the same mutation could result in an active or inactive prediction. Additionally, in some cases, mutations involving two specific amino acids produced opposite activity outcomes. For instance, mutating threonine to tyrosine in peptides associated with the MHC allele HLA-DRB10301 resulted in a shift from active to inactive. Conversely, the reverse mutation (tyrosine to threonine) for the same MHC allele resulted in a prediction change from inactive to active. These results, combined with the lack of pronounced ΔER values, suggest a synergistic interplay among multiple positions and distinct amino acids. This finding implies that the model was integrating additional features by identifying patterns and making decisions based on a more comprehensive evaluation.

As previously established, T-cell response is a highly complex process with various factors contributing. Consequently, our approach had certain limitations. One aspect was that the data was assessed by different assay formats, which was not explicitly accounted for when building the model. This also applies to other assay-specific details that were not considered. Additionally, the T-cell proliferation dataset was highly imbalanced, which may have affected the model’s ability to accurately predict inactive samples. However, this limitation was also due to the shortcomings in publicly available databases containing T-cell assay data. Furthermore, this study utilized as many MHC alleles as possible to provide users with a broader applicability. However, it is important to note that the frequency of MHC alleles varies across populations, with some alleles being more prevalent than others. This imbalance was also reflected in our dataset and may have influenced the prediction accuracy for less common MHC alleles.

According to the authors of ProtBert, fine-tuning the ProtBert model has shown to provide higher accuracy in certain tasks compared to using the embedding features. This approach could potentially enhance our model’s performance as well. Additionally, overparametrized models have been shown to improve model generalizability [28, 29], suggesting that more complex BERT models may offer a better outcome. Hence, future work could focus on fine-tuning large models, as well as utilizing the more complex BERT models. Furthermore, incorporating additional assay-related information into the model may enhance predictive accuracy. Moreover, given that our results suggest a synergistic interplay among multiple factors, a subsequent next step could be to investigate these interactions further.

## Conclusion

To explore various approaches for building IFNγ release models, different representations were used to describe peptide sequences and MHC-II alleles pseudo-sequences. In addition, feature importance and virtual single amino acid mutations were performed to gain insights into the model’s decision-making process and to improve model interpretability. The model was tested for its generalizability beyond IFNγ release by predicting T-cell proliferation, a process closely associated with IFNγ release. The findings from this study highlight the model’s applicability and its potential to support the evaluation of a T-cell activity closely related to IFNγ release.

**Key Points**

- Investigated different approaches for developing IFNγ release models.
- Evaluated the model’s generalizability to a T-cell activity closely associated with IFNγ release.
- Conducted feature importance analysis and virtual amino acid mutations to enhance model interpretability.

## Supplementary Material

SFigure1_bbaf101

SFigure2_bbaf101

supplementarytable_1_bbaf101

supplementarytable_2_bbaf101

supplementarytable_3_bbaf101

supplementary_information_fv_bbaf101

supplementary_information_fv

Figure_mutation_neg2_bbaf101

Figure_mutation_neg3_bbaf101

Figure_mutation_neg13_bbaf101

Figure_mutation_neg14_bbaf101

Figure_mutation_pos2_bbaf101

Figure_mutation_pos3_bbaf101

Figure_mutation_pos14_bbaf101

## Data Availability

Data and source code utilized in this work are available at: https://github.com/PharminfoVienna/IFNg_models.
