# Supplementary figures and images for: Exploring diverse approaches for predicting interferon-gamma release: utilizing MHC class II and peptide sequences
Source: Brief Bioinform. 2025 Mar 11;26(2):bbaf101. doi: 10.1093/bib/bbaf101 (PMC11894801; doi:10.1093/bib/bbaf101)

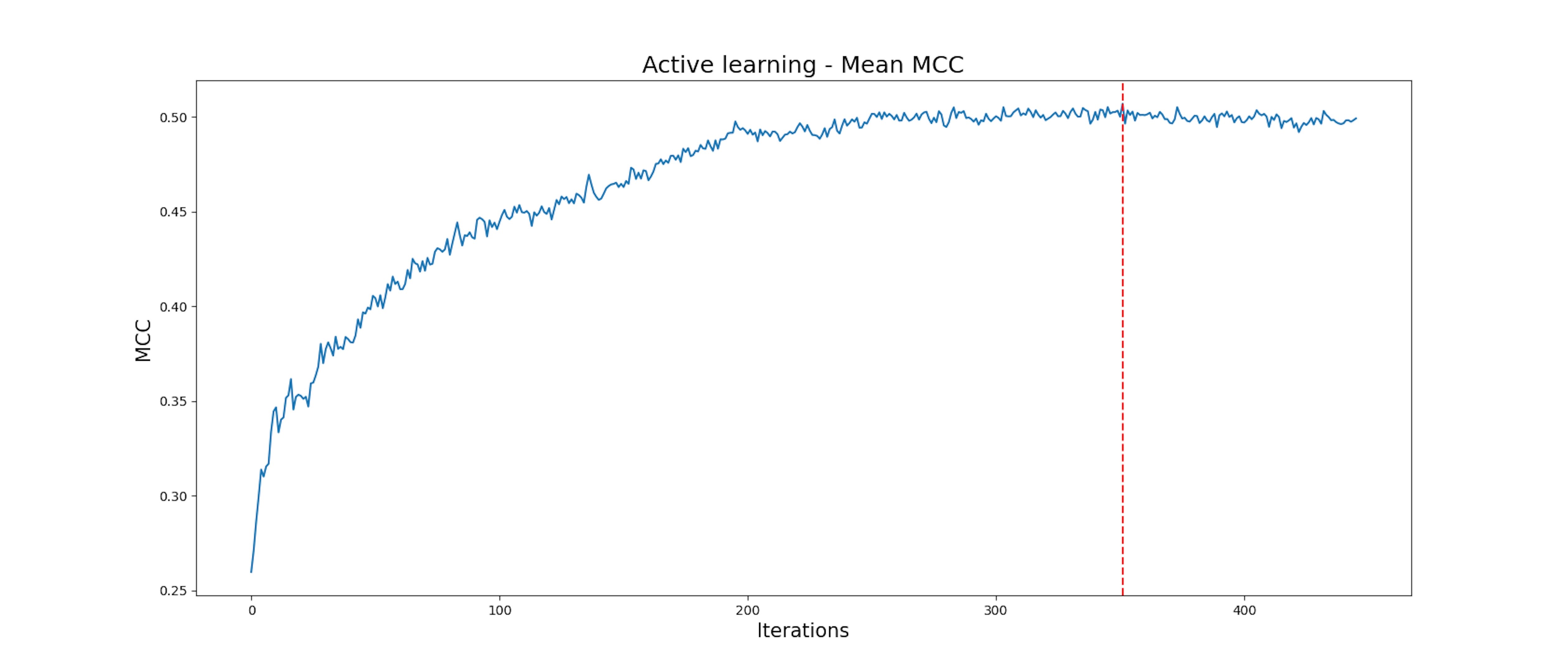

Supplement: SFigure1_bbaf101 [file sfigure1_bbaf101.jpeg]

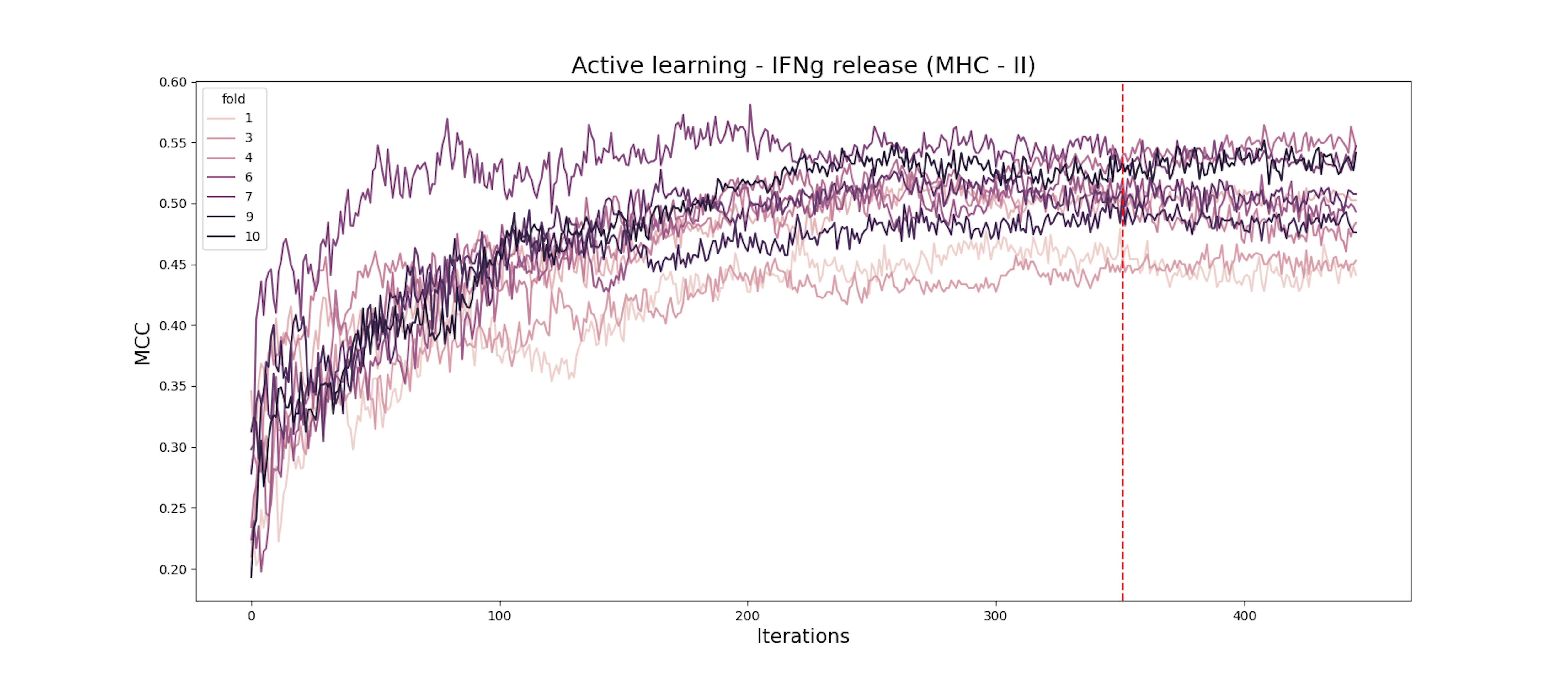

Supplement: SFigure2_bbaf101 [file sfigure2_bbaf101.jpeg]

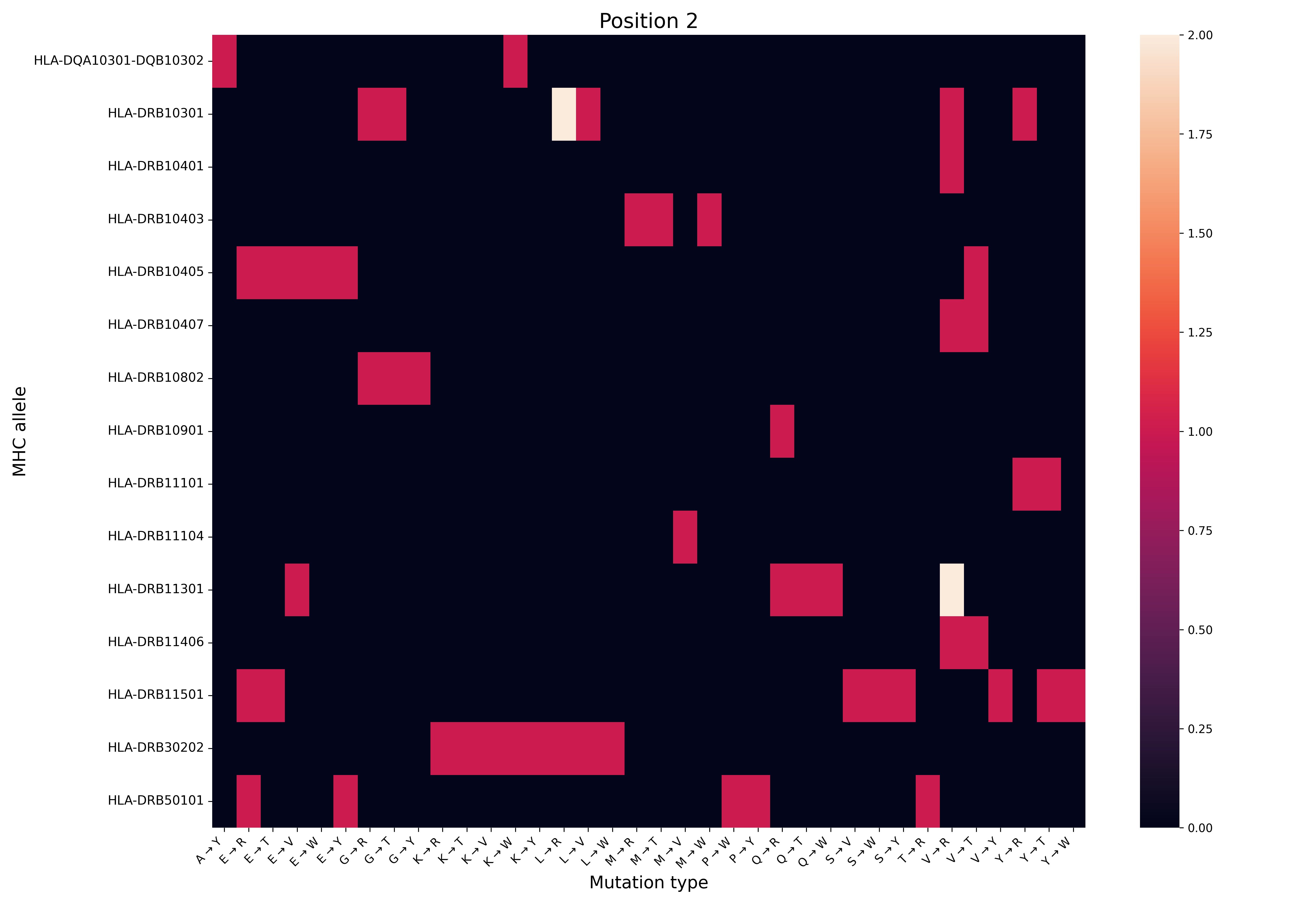

Supplement: Figure_mutation_neg2_bbaf101 [file figure_mutation_neg2_bbaf101.jpeg]

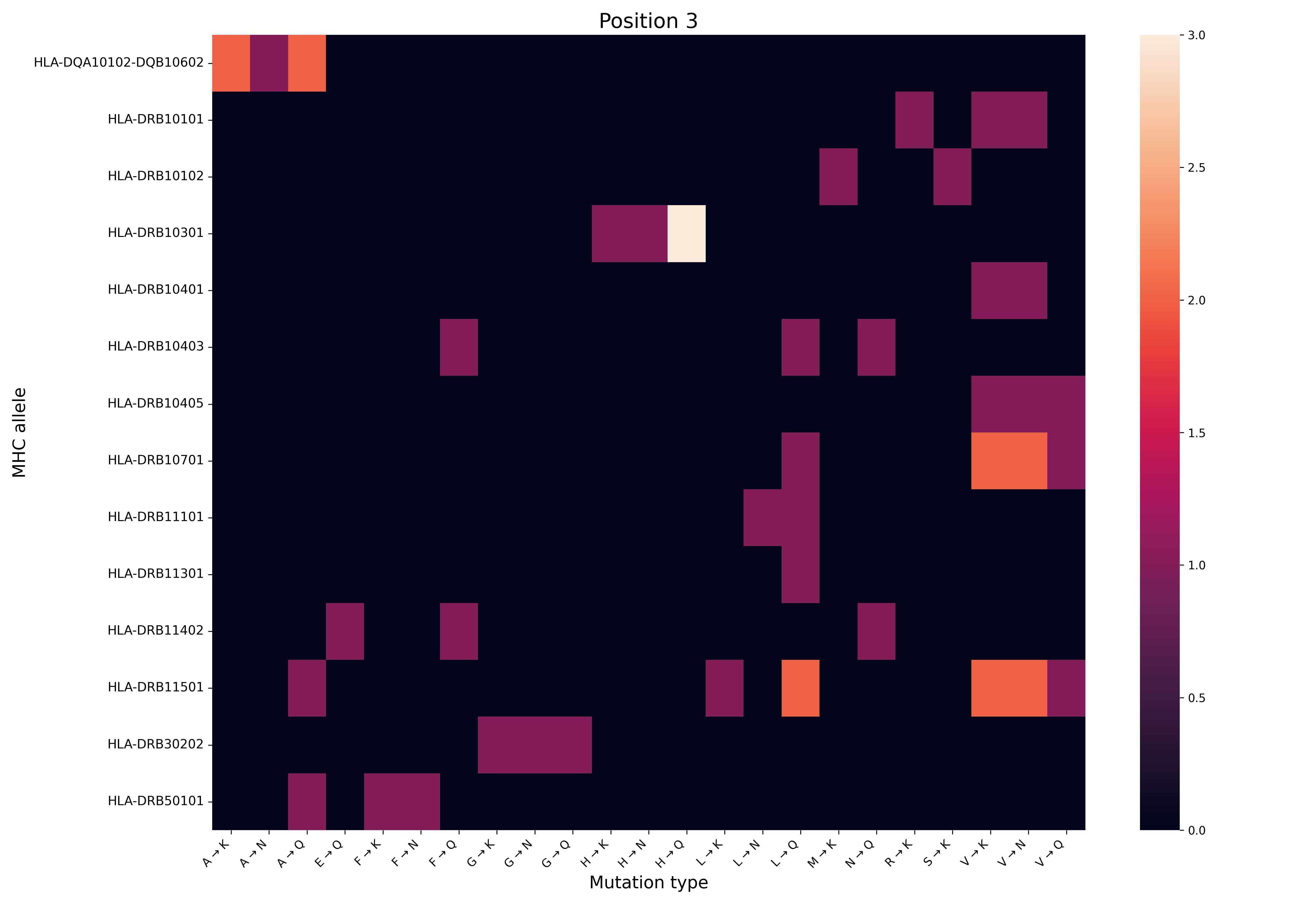

Supplement: Figure_mutation_neg3_bbaf101 [file figure_mutation_neg3_bbaf101.jpeg]

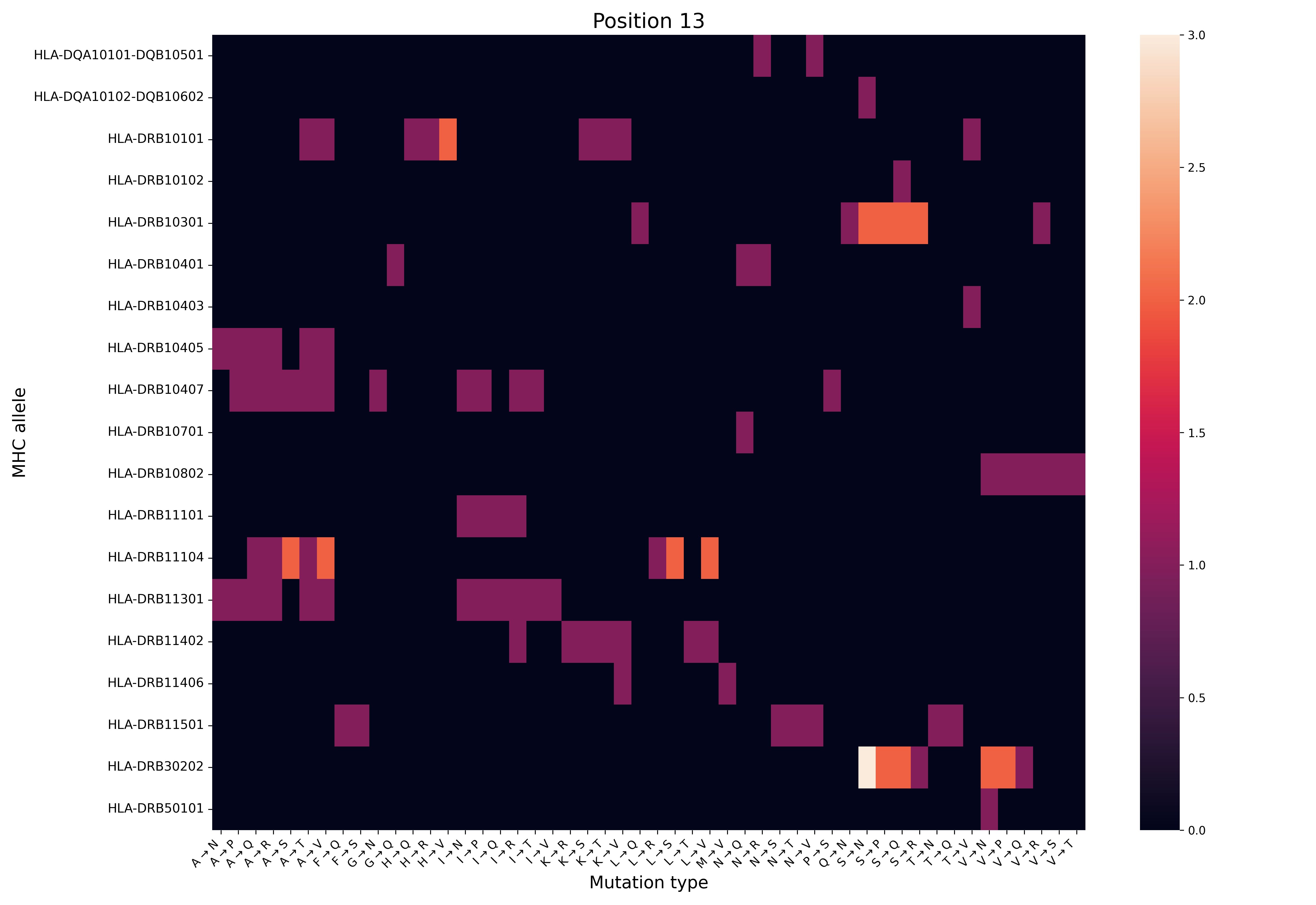

Supplement: Figure_mutation_neg13_bbaf101 [file figure_mutation_neg13_bbaf101.jpeg]

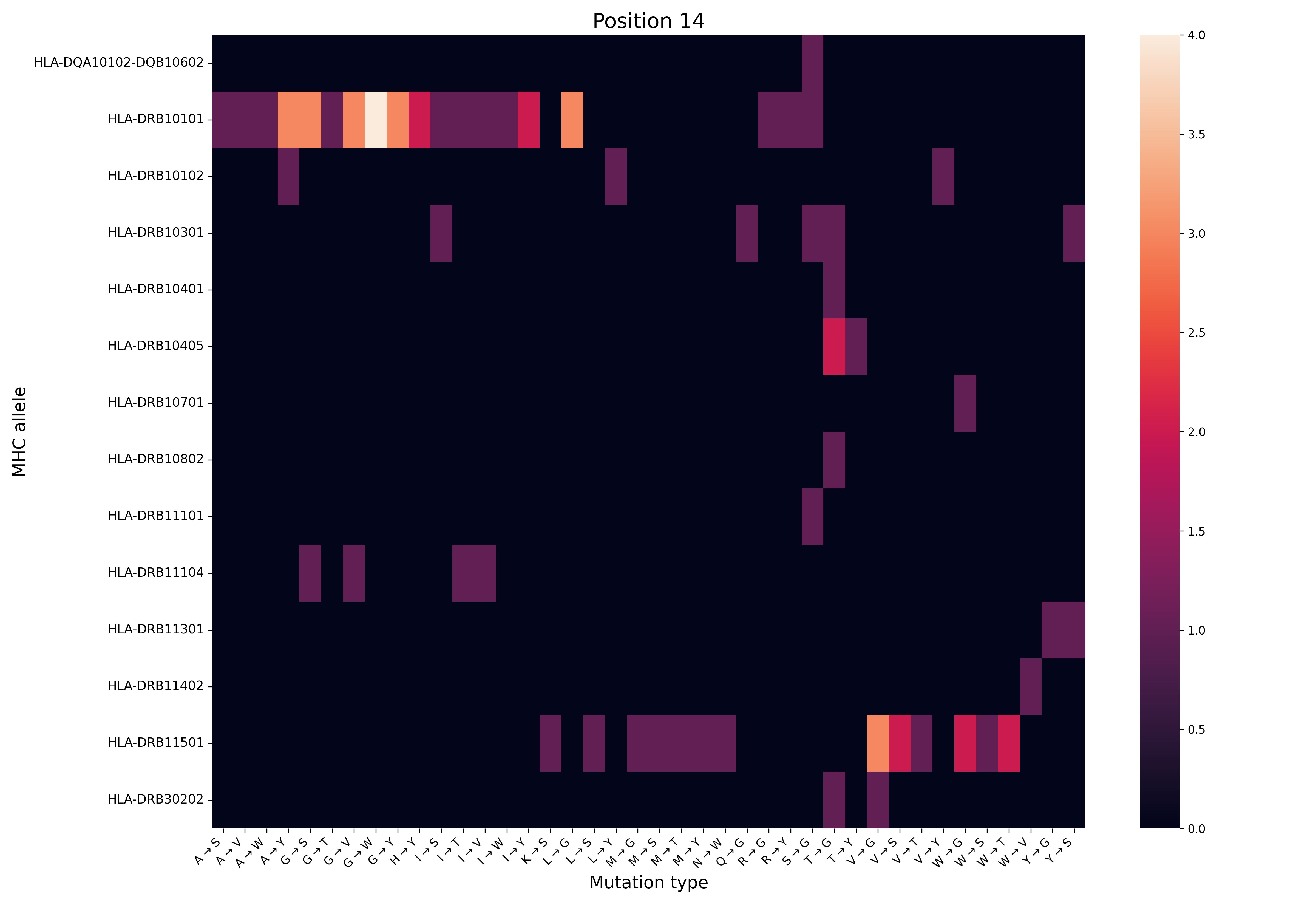

Supplement: Figure_mutation_neg14_bbaf101 [file figure_mutation_neg14_bbaf101.jpeg]

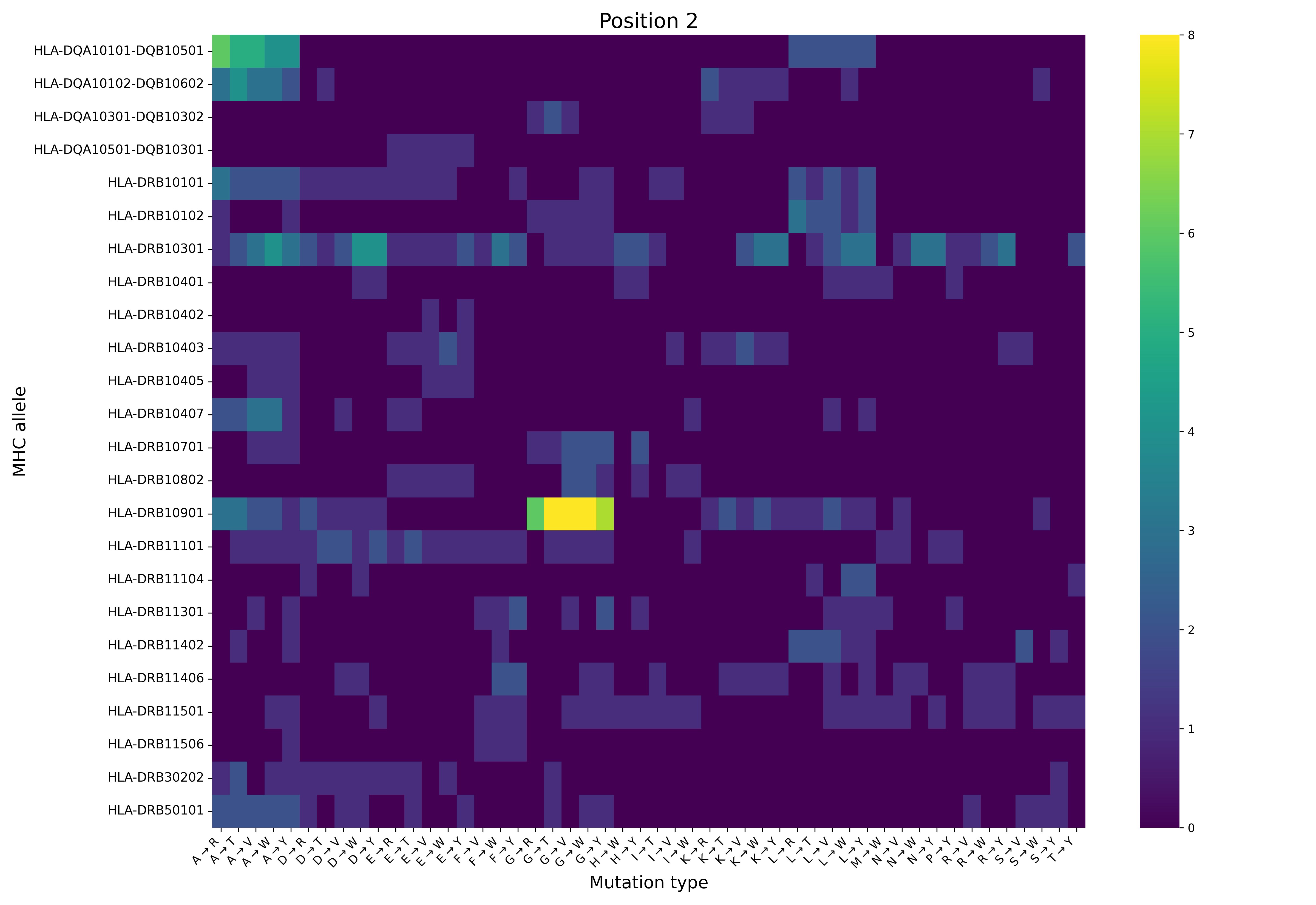

Supplement: Figure_mutation_pos2_bbaf101 [file figure_mutation_pos2_bbaf101.jpeg]

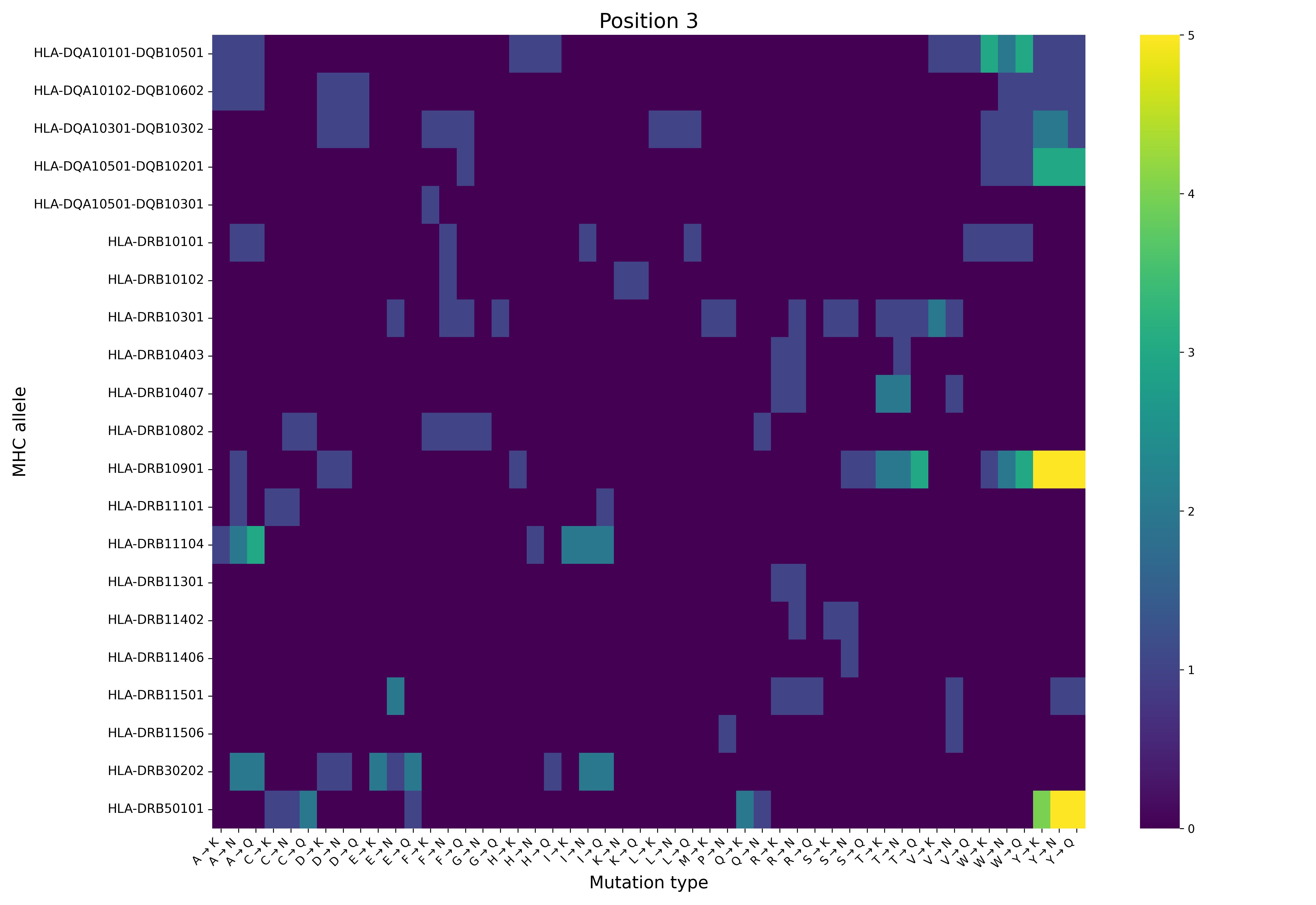

Supplement: Figure_mutation_pos3_bbaf101 [file figure_mutation_pos3_bbaf101.jpeg]

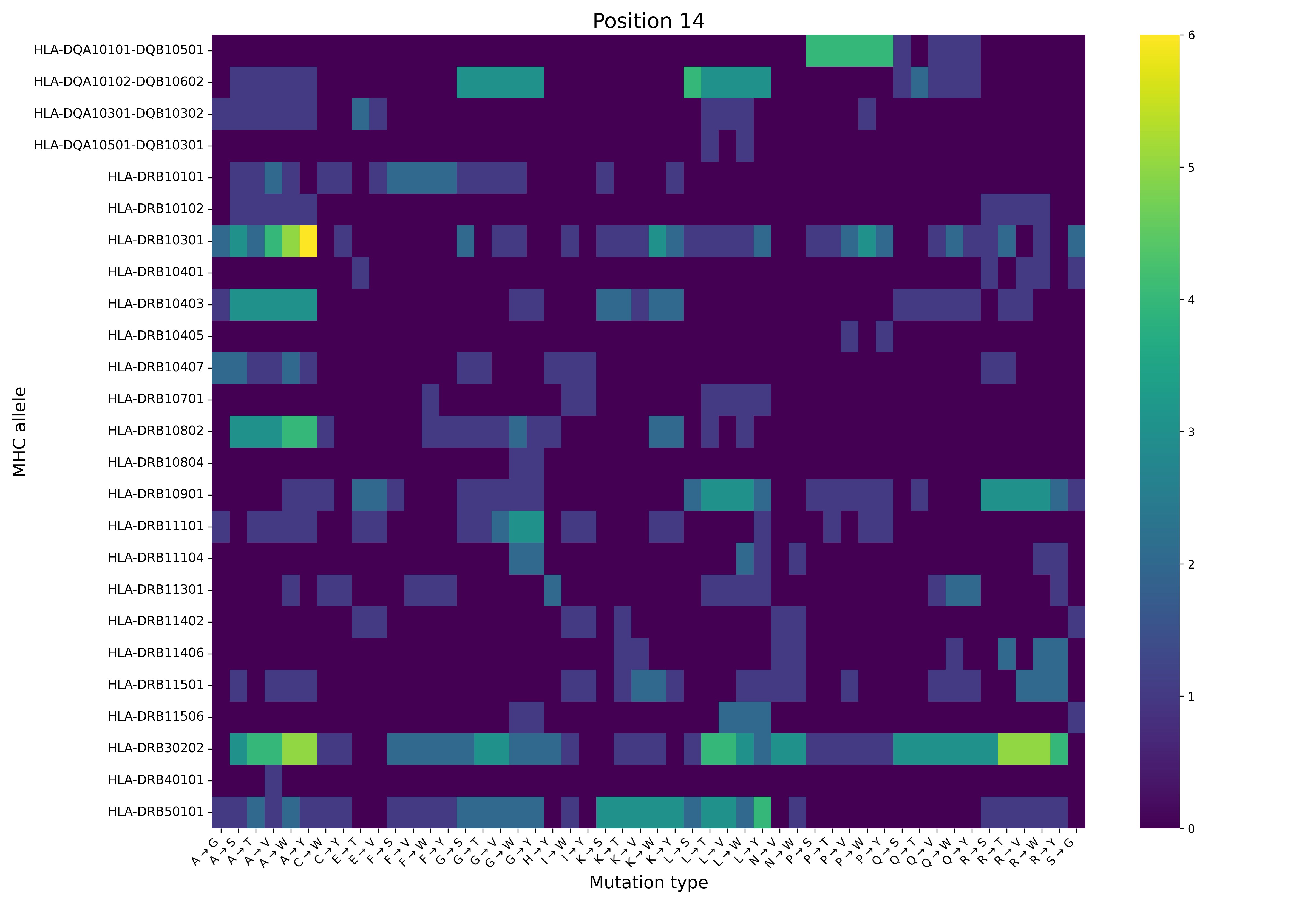

Supplement: Figure_mutation_pos14_bbaf101 [file figure_mutation_pos14_bbaf101.jpeg]
