# Supplementary material for: Exploring diverse approaches for predicting interferon-gamma release: utilizing MHC class II and peptide sequences
Source: Brief Bioinform. 2025 Mar 11;26(2):bbaf101. doi: 10.1093/bib/bbaf101 (PMC11894801; doi:10.1093/bib/bbaf101)
Supplement: supplementarytable_1_bbaf101 [file supplementarytable_1_bbaf101.docx]

| **parameters** | **values** |
| --- | --- |
| n_estimators | [100, 311, 522, 733, 944, 1155, 1366, 1577, 1788, 2000] |
| max_features | [‘auto’, ’sqrt’] |
| max_depth | [10, 20, 30, 40, 50, 60, 70, 80, 90, 100, 110] |
| min_samples_split | [2, 5, 8, 11, 14, 17, 20, 23, 26, 29, 32] |
| min_samples_leaf | [1, 4, 7, 10, 13, 16, 19, 22, 25, 28, 32] |
| bootstrap | [True, False] |
| n_iteration | 100 |
| cv | 10 |
