# Supplementary material for: Exploring diverse approaches for predicting interferon-gamma release: utilizing MHC class II and peptide sequences
Source: Brief Bioinform. 2025 Mar 11;26(2):bbaf101. doi: 10.1093/bib/bbaf101 (PMC11894801; doi:10.1093/bib/bbaf101)
Supplement: supplementarytable_2_bbaf101 [file supplementarytable_2_bbaf101.docx]

| **parameters** | **values** |
| --- | --- |
| C | [2, 6, 10] |
| gamma | [0.1, 0.5, 1] |
| kernel | [‘linear’,’rbf’] |
| n_iteration | 100 |
| cv | 10 |
