# Supplementary material for: Exploring diverse approaches for predicting interferon-gamma release: utilizing MHC class II and peptide sequences
Source: Brief Bioinform. 2025 Mar 11;26(2):bbaf101. doi: 10.1093/bib/bbaf101 (PMC11894801; doi:10.1093/bib/bbaf101)
Supplement: supplementarytable_3_bbaf101 [file supplementarytable_3_bbaf101.docx]

| Method | Algorithm | Balanced accuracy | | Matthews  correlation  coefficient | | Precision | | Recall | | Specificity | |
| --- | --- | --- | --- | --- | --- | --- | --- | --- | --- | --- | --- |
| LBE | SVM | 0.72 (0.01) | | 0.43 (0.03) | | 0.82 (0.01) | | 0.81 (0.02) | | 0.62(0.03) | |
| LBE | GBM | 0.75 (0.01) | | 0.48 (0.03) | | 0.85 (0.01) | | 0.79 (0.02) | | 0.71 (0.02) | |
| LBE | RF | 0.78 (0.01) | | 0.53 (0.02) | | 0.88 (0.01) | | 0.78 (0.02) | | 0.77 (0.03) | |
| Z-scale | SVM | 0.74 (0.02) | | 0.48 (0.04) | | 0.83 (0.02) | | 0.78 (0.03) | | 0.71 (0.03) | |
| Z-scale | GBM | 0.75 (0.02) | | 0.49 (0.04) | | 0.85 (0.02) | | 0.74 (0.03) | | 0.77 (0.03) | |
| Z-scale | RF | 0.76 (0.02) | | 0.50 (0.04) | | 0.86 (0.01) | | 0.74 (0.03) | | 0.78 (0.03) | |
| ProtBert | SVM | 0.68 (0.01) | | 0.37 (0.02) | | 0.80 (0.01) | | 0.79 (0.02) | | 0.58 (0.03) | |
| ProtBert | GBM | 0.76 (0.02) | | 0.49 (0.04) | | 0.87 (0.01) | | 0.75 (0.02) | | 0.76 (0.03) | |
| ProtBert | RF | 0.76 (0.01) | 0.50 (0.03) | | 0.89 (0.01) | | 0.72 (0.03) | | 0.81 (0.03) | |  |
