# Supplementary material for: Exploring diverse approaches for predicting interferon-gamma release: utilizing MHC class II and peptide sequences
Source: Brief Bioinform. 2025 Mar 11;26(2):bbaf101. doi: 10.1093/bib/bbaf101 (PMC11894801; doi:10.1093/bib/bbaf101)
Supplement: supplementary_information_fv_bbaf101 [file supplementary_information_fv_bbaf101.docx]

# Supplementary Information for Exploring Diverse Approaches for Predicting Interferon-Gamma Release: Utilizing MHC Class II and Peptide Sequences

Abir Omran^1^, Alexander Amberg^2^, Gerhard F. Ecker^1^,

*^1^ University of Vienna, Department of Pharmaceutical Sciences, Josef-Holaubek-Platz 2, Vienna, Austria*

*^2^ Sanofi, Preclinical Safety, Frankfurt, Germany*

Table of Contents

[Supplementary Information for Exploring Diverse Approaches for Predicting Interferon-Gamma Release: Utilizing MHC Class II and Peptide Sequences 1](#_Toc187242043)

[Supplementary Information for Comparison of Algorithms 3](#_Toc187242044)

[Hyperparameter optimization 3](#_Toc187242045)

[Performance Result 4](#_Toc187242046)

[Supplementary Information for Active Learning Result 5](#_Toc187242047)

[Supplementary Information for Virtual Amino Acid Mutation Result 6](#_Toc187242048)

[Positive to Negative Predictions 6](#_Toc187242049)

[Negative to Positive Predictions 8](#_Toc187242050)

## Supplementary Information for Comparison of Algorithms

### Hyperparameter optimization

*Supplementary Table 1: The table presents the hyperparameters used for the random search for the random forest (RF) and gradient boosting machine (GBM) models.*

[*Supplementary Table 1 here]*

*Supplementary Table 2: The table presents the hyperparameters used for the random search for the support vector machine (SVM) models.*

[*Supplementary Table 2 here]*

### Performance Result

*Supplementary Table 3: The models presented in this table include the support vector machine (SVM), gradient boosting machine (GBM), and random forest (RF) models, utilizing the three different representations used in this work. The standard deviation is presented in parentheses.*

[*Supplementary Table 3 here]*

## Supplementary Information for Active Learning Result

[ Supplementary Figure 1]

Supplementary Figure 1: This figure visualises the performance of the active learning approach across each iteration. A 10-fold cross-validation was performed, and the mean Matthews correlation coefficient (MCC) was calculated and plotted. The final model was selected by identifying the iteration with the highest mean MCC. The vertical red line indicates the iteration corresponding to the final model, which was 351.

[ Supplementary Figure 2]

Supplementary Figure 2: This figure visualises the performance of the active learning approach across each iteration. A 10-fold cross-validation was performed, and the Matthews correlation coefficient for all folds at each iteration was plotted. The vertical red line indicates the iteration corresponding to the final model, which was 351.

## Supplementary Information for Virtual Amino Acid Mutation Result

### Positive to Negative Predictions

[ Supplementary Figure 3]

Supplementary Figure 3: The figure visualises the top 50 mutations at position 2 for the active data points that resulted in a changed prediction, showing the count for each mutation type for each given allele.

[ Supplementary Figure 4]

Supplementary Figure 4: The figure visualises the top 50 mutations at position 3 for the active data points that resulted in a changed prediction, showing the count for each mutation type for each given allele.

[ Supplementary Figure 5]

Supplementary Figure 5: The figure visualises the top 50 mutations at position 13 for the active data points that resulted in a changed prediction, showing the count for each mutation type for each given allele.

[ Supplementary Figure 6]

Supplementary Figure 6: The figure visualises the top 50 mutations at position 14 for the active data points that resulted in a changed prediction, showing the count for each mutation type for each given allele.

### Negative to Positive Predictions

[ Supplementary Figure 7]

Supplementary Figure 7: The figure visualises the top 50 mutations at position 2 for the inactive data points that resulted in a changed prediction, showing the count for each mutation type for each given allele.

[ Supplementary Figure 8]

Supplementary Figure 8: The figure visualises the top 50 mutations at position 3 for the inactive data points that resulted in a changed prediction, showing the count for each mutation type for each given allele.

[ Supplementary Figure 9]

Supplementary Figure 9: The figure visualises the top 50 mutations at position 13 for the inactive data points that resulted in a changed prediction, showing the count for each mutation type for each given allele.

[ Supplementary Figure 10]

Supplementary Figure 10: The figure visualises the top 50 mutations at position 14 for the inactive data points that resulted in a changed prediction, showing the count for each mutation type for each given allele.
